# Effects of troxerutin on inflammatory cytokines and BDNF levels in male offspring of high-fat diet fed rats

**DOI:** 10.22038/AJP.2019.13587

**Published:** 2019

**Authors:** Maryam Hoseindoost, Mohammad Reza Alipour, Fereshteh Farajdokht, Roghayeh Diba, Parvin Bayandor, Keyvan Mehri, Sepehr Nayebi Rad, Shirin Babri

**Affiliations:** 1 *Drug Applied Research Center, Tabriz University of Medical Sciences, Tabriz, Iran*; 2 *Tuberculosis and Lung Disease Research Center, Tabriz University of Medical Sciences, Tabriz, Iran*; 3 *Student Scientific Research Center, Tehran University of Medical Sciences, Tehran, Iran*

**Keywords:** Troxerutin, Maternal high fat diet, BDNF, Inflammatory cytokines

## Abstract

**Objective:**

In the present study, we aimed to examine the effect of troxerutin treatment on levels of brain-derived neurotrophic factor (BDNF), and tumor necrosis factor-alpha (TNF-α) and interleukin-6 (IL-6), as pro-inflammatory cytokines, in the blood and hippocampus of high-fat diet (HFD) fed dams and their male offspring.

**Materials and Methods:**

Forty virgin female Wistar rats, 3 weeks old, were divided into two groups (n=20/per group) and fed control diet (CD), or HFD for 8 weeks. After mating, pregnant animals were assigned to four subgroups including: control (CD), control+troxerutin (CD+T), high-fat diet (HFD), and HFD+troxerutin (HFD+T) groups. HFD was continued during pregnancy and lactation. Troxerutin (150 mg/kg/day, P.O.) was administered during pregnancy in the CD+T and HFD+T groups. On postnatal day (PND) 21, male offspring were separated and fed a normal diet until PND 90. Inflammatory cytokines (TNF-α, and IL-6) and BDNF levels were measured in the serum and hippocampal samples using rat-specific enzyme-linked immunosorbent assay (ELISA) kits.

**Results:**

Our findings showed a significant increase in the serum levels of TNF-α and IL-6, but a decrease in BDNF levels in the serum of HFD-fed dams in comparison with CD group, which was reversed by troxerutin. Moreover, troxerutin treatment, during pregnancy, significantly decreased TNF-α and IL-6 levels, but increased BDNF level in the serum and hippocampus of HFD+T offspring in comparison with HFD offspring.

**Conclusion:**

These results showed that troxerutin could prevent the harmful effects of maternal HFD on their offspring through inhibition of pro-inflammatory cytokines and elevation of BDNF levels.

## Introduction

High-fat diet (HFD) is one of the important issues that affect the health of the society. A growing body of evidence suggests that overconsumption of HFD during the prenatal and early postnatal periods, plays a significant role in the development of offspring (Benkalfat et al., 2011; Parente et al., 2008). 

It was emphasized that HFD could increase the risk of several diseases such as type 2 diabetes, metabolic syndrome, and neurological disorders in offspring (Ashino et al., 2012; Giriko et al., 2013; Sasaki et al., 2013). Overconsumption of HFD is also associated with chronic inflammatory responses and macrophage accumulation in the adipose tissue (Madan et al., 2009; Zhou and Pan, 2015). Moreover, HFD activates cellular stress signaling and increases the production of circulatory inflammatory mediators in the hippocampus which in turn, results in cognitive dysfunction and hippocampal-dependent memory impairment in offspring (Pistell et al., 2010). The hippocampus is one of the critical brain structures that is associated with learning and memory processing (Noble et al., 2014). It has been reported that HFD could decline the hippocampal neurogenesis and elevate oxidative stress during the postnatal development of offspring (Molteni et al., 2002; Tozuka et al., 2009).

Brain-derived neurotrophic factor (BDNF) has a crucial role in regulating hippocampal neurogenesis and differentiation, and higher levels of BDNF is associated with memory improvement (Erickson et al., 2010; Taliaz et al., 2010). BDNF is also known as an anorexigenic factor which regulates food intake and central energy balance (Pelleymounter et al., 1995). 

Troxerutin, known as vitamin p4, is found in coffee, tea, cereal grains, and a variety of fruits and vegetables. Troxerutin possesses many pharmacological and biological effects such as anti-oxidative, anti-inflammatory, and anti-diabetic properties (Chung et al., 2005; Fan et al., 2009; Farajdokht et al., 2017). It was also reported that troxerutin has neuroprotective effects on the hippocampus in high-cholesterol diet fed mice (Lu et al., 2011). Lately, our group has shown that troxerutin attenuated anxiety- and depressive-like behaviors (Bayandor et al., 2018) and also improved maternal HFD-induced spatial memory impairments in the offspring of the HFD-fed dams (Diba et al., 2018). Therefore, according to the beneficial impacts of troxerutin, we aimed to examine its effects on BDNF and inflammatory cytokines in the blood and hippocampus of male offspring of HFD-fed dams.

## Materials and Methods

**Animals**

Forty female Wistar rats, 3 weeks old, were purchased from the animal house of Tabriz University of Medical Sciences. Animals were housed three per cage under standard conditions with 12 hr light/dark cycles (lights were on at 6:00 AM), at 20-22°C; with 45-55% humidity, and had free access to food and water. All experimental procedures were conducted in accordance with the instruction for the care and use of laboratory animals prepared by Tabriz University of Medical Sciences and approved by the Regional Ethics Committee of Tabriz University of Medical Sciences.

**Experimental groups**

Before mating, animals were randomly divided into two groups (n=20/group) and fed control diet (CD), or high-fat diet (HFD) for 8 weeks. Mating was performed by housing of the females with adult males overnight. After confirmation of pregnancy by examining vaginal smears for the presence of sperm, pregnant rats were kept in individual cages at room temperature (24±1°C). Pregnant rats were assigned to four subgroups (10 animals in each group) as follows: CD (received a control diet), CD+T (received a control diet and treated with troxerutin 150 mg/kg/day), HFD (received a HFD), and HFD+T (received a HFD and troxerutin 150 mg/kg/day). High-fat diet feeding in the related groups was continued throughout the lactation. Animals in the troxerutin-treated groups were treated with troxerutin (150 mg/kg/day per os (P.O.)) (Merck, Germany) during pregnancy. The male offspring were weaned on postnatal day (PND) 21 and kept in their respective maternal groups. All offspring were fed a control diet until PND 90. Body weight of dams were measured before pregnancy and body weight of offspring was measured on PND 90.

**Sampling and assessments**

At the end of the experiments, animals were anesthetized using ketamine (80 mg/kg, intraperitoneal (i.p.)) and xylazine (12 mg/kg, i.p.). Blood samples were collected from the heart of offspring. Maternal blood samples were collected once before mating and then at the end of lactation. All blood samples were centrifuged at 4000 rpm for 15 min, and serum was separated and kept at -70ºC for measurement of TNF-α, IL-6, and BDNF levels. After decapitation, the hippocampus was carefully isolated and hippocampal extracts were prepared in lysis buffer (137 mM NaCl, 20 mM Tris –HCl (pH 8.0), 1% NP-40, 10% glycerol, 1 mM phenylmethylsulfonyl fluoride, 10 µg/ml aprotinin, 1 µg/ml leupeptin, and 0.5 mM sodium vanadate). Homogenates were centrifuged at 12,000 rpm for 20 min at 4°C to remove insoluble materials. Then, the supernatants were collected and kept at -70°C for later measurements. Rat-specific ELISA kits (EASTBIOPHARM, China) were used to measure IL-6, TNF-α, and BDNF levels in the blood and hippocampus according to the manufacturer's protocols.

**Statistical analysis**

The data are expressed as mean±SEM. Statistical analysis was performed using SPSS software version 16. Paired sample t-test was used to compare maternal parameters before and after pregnancy, and the t-student test was used to compare differences between maternal CD and HFD groups. Moreover, one-way ANOVA followed by Tukey *post-hoc* test was used for analysis of data among offspring groups. The statistical significance level was set at p<0.05. 

## Results

**Effects of HFD and troxerutin on maternal serum pro-inflammatory cytokines (TNF-α and IL-6) and BDNF levels**

**Figure 1:**
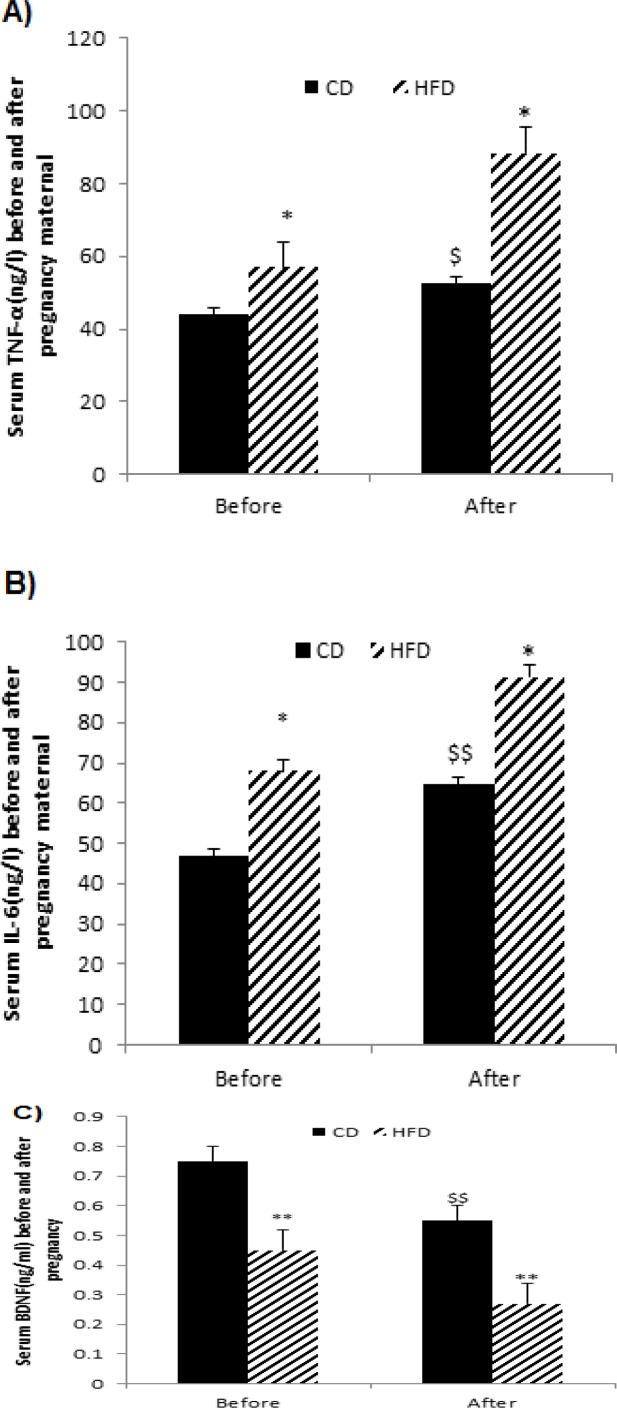
Effects of HFD on maternal serum inflammatory cytokines (TNF-α and IL-6) and BDNF levels before pregnancy and after pregnancy. Values are expressed as Mean±SEM (n=8 per groups). Paired sample t-test was used to compare maternal parameters before and after pregnancy; *p<0.05, and **p<0.01 indicate the difference in the CD and HFD groups before and after pregnancy. t-student test; ^$^p<0.05, and ^$$^p<0.01 indicate the difference between CD and HFD groups [CD: Control Diet, CD+T: Control Diet+Troxerutin, HFD: High-Fat Diet, and HFD+T: High Fat Diet+Troxerutin]

Statistical analysis demonstrated that HFD significantly reduced serum BDNF level but increased TNF-α and IL-6 concentrations during pre-pregnancy as compared with the CD group (p<0.01, Figure 1). Moreover, the results of paired-sample t-test showed that HFD significantly increased inflammatory cytokines (TNF-α and IL-6) but decreased BDNF levels at the end of pregnancy in comparison with the pre-pregnancy (Figure 1). 

**Figure 2:**
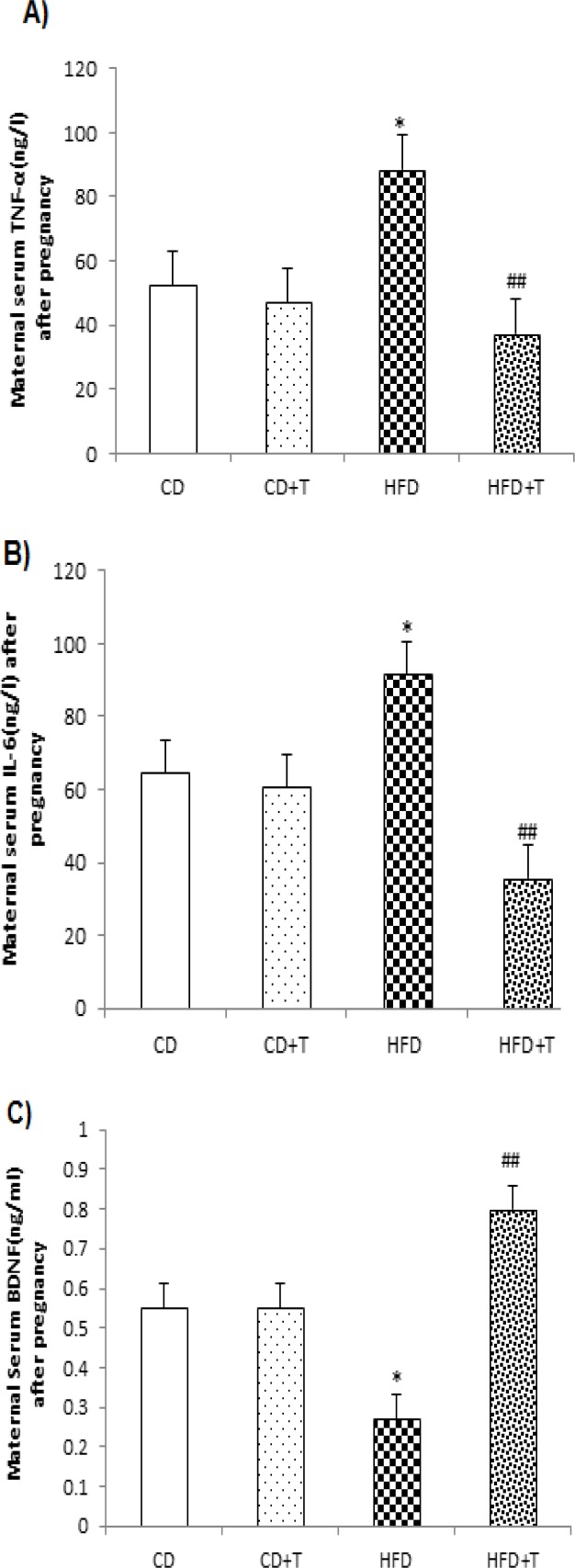
Effect of troxerutin treatment on maternal TNF-α (A), IL-6 (B) and BDNF (C) serum levels. Values are expressed as Mean±SEM (n=8 per groups). One-way ANOVA followed by Tukey *post-hoc* test was used for making comparisons. *p<0.05 compared with CD group, and ##p<0.01 compared with HFD group. [CD: Control Diet, CD+T: Control Diet+Troxerutin, HFD: High Fat Diet, and HFD+T: High Fat Diet+Troxerutin]

Moreover, HFD significantly (p<0.05) increased maternal serum TNF-α and IL-6 levels but decreased BDNF levels after pregnancy as compared to the control diet-received animals. However, troxerutin administration in the HFD+T group during pregnancy, caused a marked (p<0.01) decrease in TNF-α and IL-6 levels, but a significant increase in BDNF levels (p<0.01, Figure 2) in comparison with HFD dams. 

**Effects of troxerutin on inflammatory cytokines (TNF-α and IL-6) and BDNF levels in the serum of offspring**

**Figure 3:**
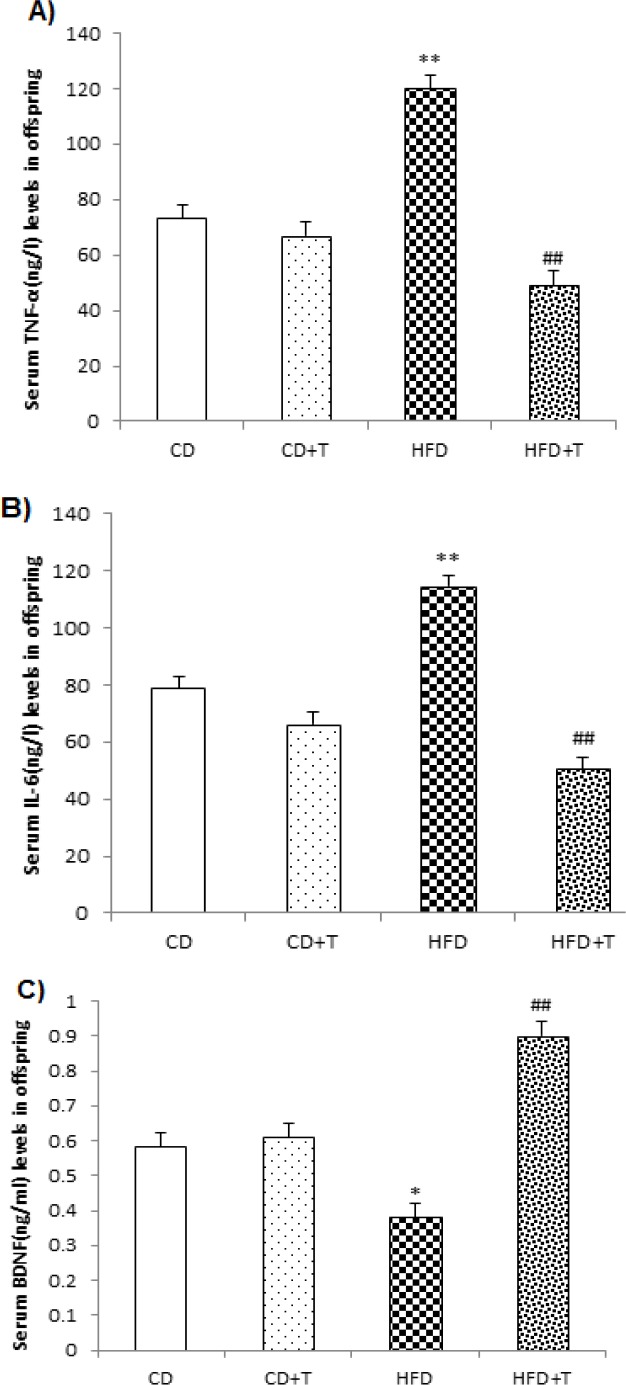
Effect of troxerutin treatment on serum concentration of TNF-α (A), IL-6 (B) and BDNF (C) in offspring. Values are expressed as Mean±SEM (n=8 per groups). One-way ANOVA followed by Tukey *post-hoc* test was used for making comparisons. *p<0.05, and **p<0.01 compared with CD group, and ##p<0.01 compared with HFD group. [CD: Control Diet, CD+T: Control Diet+Troxerutin, HFD: High Fat Diet, and HFD+T: High Fat Diet+Troxerutin]

Results of offspring indicated that HFD significantly (Figure 3, p<0.01) increased serum levels of TNF-α and IL-6 but decreased BDNF levels (p<0.05) as compared to the CD group. Nevertheless, troxerutin treatment during pregnancy caused a significant decrease in serum levels of TNF-α and IL-6 but increased BDNF levels in HFD+T group offspring compared with the HFD and control offspring groups (p<0.01, Figure 3). 

**Effects of troxerutin on inflammatory cytokines (TNF-α and IL-6) and BDNF levels in the hippocampus of offspring**

**Figure 4:**
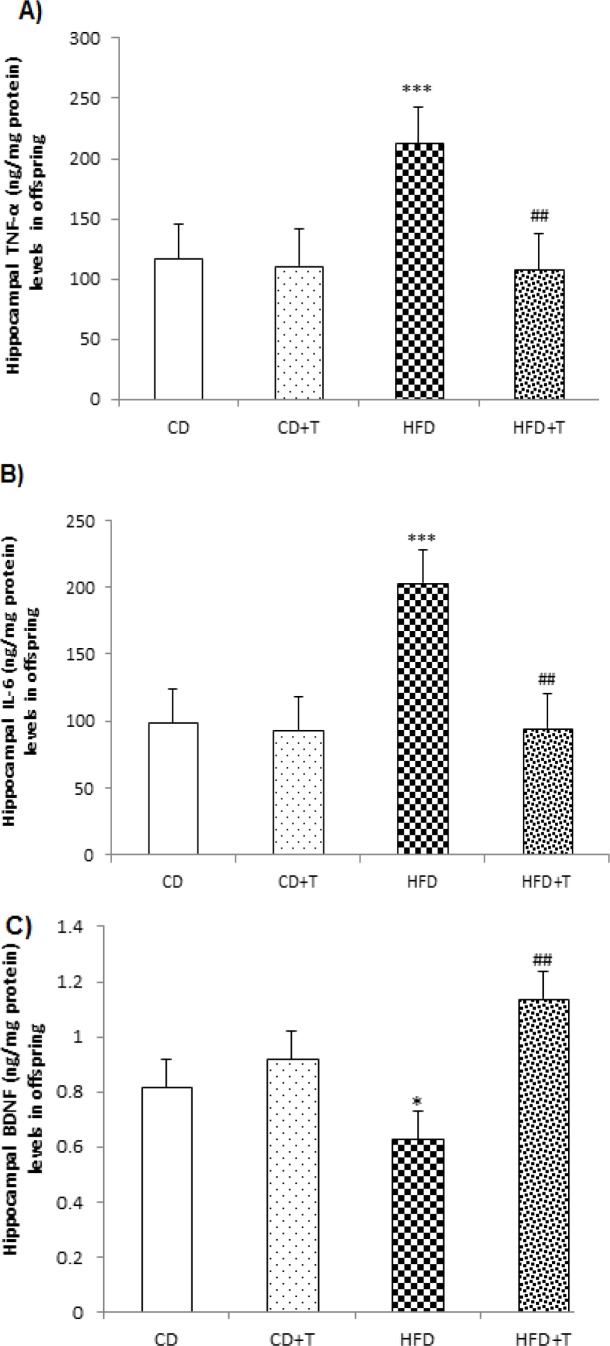
Effect of troxerutin treatment on hippocampal TNF-α (A), IL-6 (B) and BDNF (C) levels in offspring. Values are expressed as Mean±SEM (n=8 per groups). One-way ANOVA followed by Tukey *post-hoc* test was used for making comparisons. *p<0.05, and ***p<0.001 compared with CD group, and ##p<0.01 compared with HFD group. [CD: Control Diet, CD+T: Control Diet+Troxerutin, HFD: High Fat Diet, and HFD+T: High Fat Diet+Troxerutin]

Our data also showed that HFD significantly (Figure 4, p<0.001) increased TNF-α and IL-6 levels in the hippocampus of HFD offspring in comparison with the CD group. Moreover, hippocampal BDNF level showed a significant (p<0.05) decrease in the HFD-fed offspring in comparison with control offspring (Figure 4C). However, troxerutin treatment during pregnancy significantly (p<0.01) decreased the inflammatory cytokines and increased BDNF levels in the hippocampus of HFD+T offspring. 

**Effects of HFD and troxerutin on body weight change in dams and offspring**

**Figure 5:**
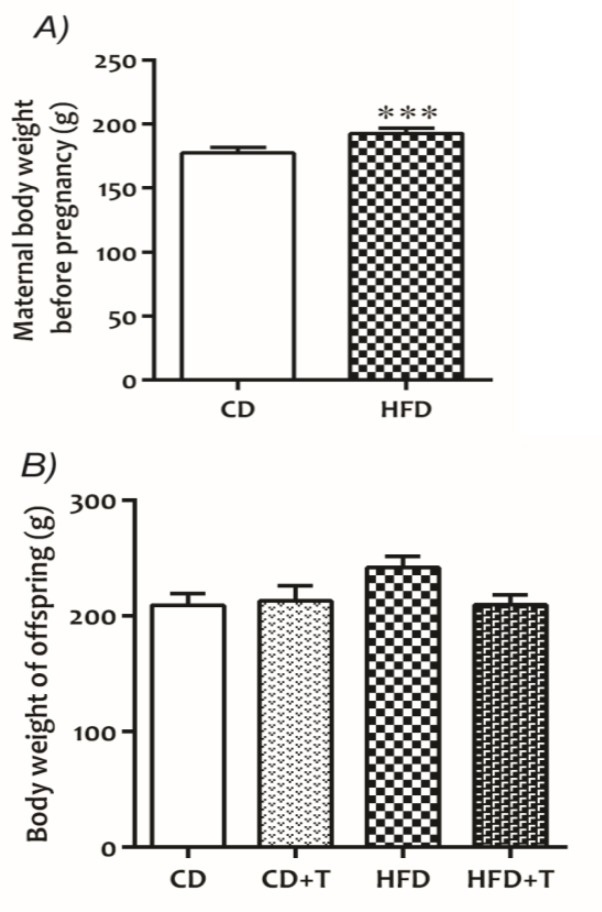
Effect of HFD and troxerutin on body weight of (A) maternal and (B) offspring. Values are expressed as Mean±SEM (n=8 per groups). Sample t-test was used for making comparisons (***p<0.001). [CD: Control Diet, CD+T: Control Diet+Troxerutin, HFD: High Fat Diet, and HFD+T: High Fat Diet+Troxerutin]

The results of sample t-test revealed that HFD significantly (p<0.001, Figure 5A) increased maternal body weight before pregnancy. However, there was no significant difference in body weight of offspring 90 days after birth among different groups (Figure 5B).

## Discussion

This study aimed to investigate the effects of chronic troxerutin treatment on BDNF and pro-inflammatory cytokines in the blood and hippocampus of high-fat diet fed pregnant rats as well as their male offspring. According to our results, HFD consumption significantly increased body weight and inflammatory cytokines, while decreased BDNF in the maternal serum before pregnancy. The same changes were observed in their respective offspring groups both in the serum and hippocampus. Although troxerutin treatment during pregnancy did not change body weight of offspring, it significantly attenuated IL-6 and TNF-α concentration, and improved BDNF levels in the HFD-fed mothers and their offspring both in the serum and hippocampus.

Previous studies showed that HFD consumption, during pregnancy and suckling, leads to hippocampal inflammation, reduces hippocampal neurogenesis and impairs long-term spatial memory in offspring (Bilbo and Tsang, 2010; Boitard et al., 2014; Boitard et al., 2012). Moreover, HFD exposure increases maternal circulating levels of the pro-inflammatory cytokines (TNF-α and IL-1β) during pregnancy and macrophage accumulation in the placenta (Ashino et al., 2012; Challier et al., 2008). Similarly, in the present study, we found that maternal HFD increased TNF-α and IL-6 in dams. Moreover, several studies showed that HFD consumption is associated with increased lipid peroxidation in the serum and brain of offspring (Molteni et al., 2002; Tozuka et al., 2009). Lipotoxicity triggers inflammatory responses and oxidative stress which in turn, suppress the brain development in offspring (Niculescu and Lupu, 2009; Tozuka et al., 2009). In the current study, troxerutin treatment in the HFD-fed group caused a significant decrease in serum level of IL-6 and TNF-α in dams, as well as in the serum and hippocampus of their offspring. According to the previous studies, troxerutin as a member of flavonoid family, has inhibitory effects on the nuclear factor-kappa B (NF-ĸB)-mediated inflammatory responses and protects various tissues against inflammation (Badalzadeh et al., 2016; Fan et al., 2009; Zhang et al., 2016). Based on these finding, we suggest that troxerutin could have a beneficial impact during pregnancy in HFD-fed group via its anti-inflammatory effects and via prevention of the increase in inflammatory cytokines in the postnatal period. 

Moreover, our data showed a significant decrease in BDNF level in the serum samples of HFD-fed dams and their offspring. In agreement with our findings, previous studies revealed that HFD reduces the hippocampal BDNF level during the early postnatal development of offspring (Molteni et al., 2002; Wu et al., 2004). BDNF as a neurotrophic factor plays an important role in the development of the nervous system during the prenatal period and later in postnatal life, and also involves in learning and memory processes (Datta et al., 2009; Souza et al., 2015). Therefore, alterations in BDNF level due to maternal HFD exposure, affect hippocampus neurogenesis and synaptic plasticity of offspring which may lead to learning and memory deficits in adolescence. In recent years, some studies demonstrated that different members of the flavonoids family could improve cognitive deficits possibly through elevation of BDNF (Komulainen et al., 2008; Macready et al., 2009). In the present study, we observed that troxerutin treatment during pregnancy significantly increased BDNF in the serum of HFD-fed dams as well as serum and hippocampus of their offspring. These findings for the first time, showed that troxerutin consumption during pregnancy exerts neuroprotective effects. Our results emphasize that the mechanism of this protective effect is partially related to inhibition of neuroinflammation in the brain and increased BDNF level. 

Overall, this investigation revealed that maternal exposure to HFD reduced BDNF levels and elevated IL-6 and TNF-α concentration in maternal serum as well as in serum and hippocampus of their male offspring. However, troxerutin treatment during pregnancy reversed these changes both in the mothers and offspring. More studies are needed to clarify the exact mechanism of the protective effect of troxerutin in this regard.
